# Supplementary material for: Modeling and Forecasting Dead-on-Arrival in Broilers Using Time Series Methods: A Case Study from Thailand
Source: Animals (Basel). 2025 Apr 20;15(8):1179. doi: 10.3390/ani15081179 (PMC12024027; doi:10.3390/ani15081179)
Supplement: Supplementary file 1 [file animals-15-01179-s001.zip › animals-3580998-supplementary.pdf]

Table S1. Forecasted monthly %DOA for broilers in 2025 based on five time series models.

Month	SARIMA	NNAR	TBATS	ETS	XGBoost
January	0.41	0.60	0.53	0.51	0.56
February	0.45	0.53	0.56	0.59	0.57
March	0.44	0.61	0.52	0.54	0.50
April	0.43	0.52	0.44	0.48	0.50
May	0.39	0.53	0.38	0.37	0.36
June	0.36	0.44	0.35	0.38	0.35
July	0.40	0.53	0.34	0.36	0.36
August	0.40	0.50	0.34	0.40	0.37
September	0.37	0.50	0.33	0.28	0.34
October	0.45	0.34	0.33	0.36	0.37
November	0.37	0.33	0.37	0.42	0.40
December	0.48	0.32	0.44	0.47	0.41

SARIMA: AutoRegressive Integrated Moving Average; NNAR: Neural Network AutoRegressive; TBATS: Trigonometric Box-Cox ARMA Trend Seasonal; ETS: Exponential Smoothing State Space; XGBoost: Extreme Gradient Boosting.
